# The mitochondrial genome of *Paralimna (Paralimna) concors* (Diptera: *Ephydridae*)

**DOI:** 10.1080/23802359.2020.1833374

**Published:** 2020-11-08

**Authors:** Chenjing Zhao, Jiaojie Wang, Bing Zhang, Jiangwan Han, Junhua Zhang, Danli Zhang, Liang Wang

**Affiliations:** a Department of Biology, Taiyuan Normal University, Jinzhong, PR China; b College of Plant Protection, China Agricultural University, Beijing, PR China; c Forestry Technology Comprehensive Service Station of Diebu, Diebu, PR China; d Institute of Animal and Plant Quarantine, Chinese Academy of Inspection and Quarantine, Beijing, PR China

**Keywords:** Mitochondrial genome, *Ephydridae*, *Paralimna* (*Paralimna*) *concors*, phylogeny

## Abstract

The mitogenome of *Paralimna (Paralimna) concors* was sequenced. The mitogenome was 16,155 bp totally, consisting of 13 protein-coding genes (PCGs), two rRNAs, and 22 transfer RNAs. The nucleotide composition biases toward A and T is 78.6％ of the entirety. All PCGs start with ATN codons except COI and ND1, and end with TAA or incomplete stop codon. Phylogenetic analyses based on 11 dipteran species supported the monophyly of Ephydroidea and the relationship of Opomyzoidea + (Ephydroidea + (Lauxanioidea + (Sphaeroceroidea + (Sciomyzoidea + Tephritoidea)))).

## Introduction

Adults of *Ephydridae* are often dull and dark colored, but unusually diverse in body structure, vestiture, and ornamentation (Mathis and Zatwarnicki 1998). The ephydrid flies with about 2000 described species from the world are placed in superfamily Ephydroidea (Pape et al. 2011). The genus *Paralimna* Loew, 1862 is one of the richest in species genera in the tribe Dryxini. The attention of many dipterologists was attracted to this group of Diptera because the largest and remarkable specimens were discovered in the field (Krivosheina and Ozerov 2020). Species of *Paralimna* regularly occur in grassy habitats (Foote 1995).

The adult specimens of *Paralimna (Paralimna) concors* (Voucher number: CAU-YDEPHY-Para-1) used for this study were collected from 2 W Hown District (19.8976°N, 101.1438°E, 1278 m), Pak Beng, Laos, on 25 Jun 2015. The specimens were identified by Liang Wang and deposited in the Entomological Museum of China Agricultural University (CAU).

The genomic DNA was extracted from adult’s whole body using the DNeasy DNA Extraction kit (TIANGEN) and stored at −20 °C refrigerator. DNA samples were pooled for next-generation sequencing library construction following the method of Gillett et al. (2014). All quantified DNA extracts were included in a single pool at equimolar concentration, aiming for 50 ng/ul of dsDNA per sample, resulting in a DNA pool of approximately 5 ug. The library was sequenced on an Illumina HiSeq 2500 by BIONONA CO., LTD. Rough read data were trimmed and cropped in Trimmomatic version 0.30 (Bolger et al. 2014) with the default setting. Four Gigabytes of high-quality reads were used to assemble mitogenomes with the *de novo* assembler IDBA_UD (Peng et al. 2012). The bait sequence COI was amplified by standard PCR reactions and BLAST search was carried out with BioEdit version 7.0.5.3 and the position of all *tRNA* genes was confirmed using tRNAscanSE version 2.0 (Lowe and Chan 2016). The nearly complete mitogenome of *Paralimna (Paralimna) concors* (MT938921) was 16,154 bp in length and consisted of 13 typical invertebrate PCGs, 22 transfer *RNA* genes, two *rRNA* genes (*12S* and *16S*), and part control region, which were similar to other Diptera flies reported before (Li et al. 2016; Zhou et al. 2017; Qilemoge et al. 2018; Ren et al. 2019). The nucleotide composition of the mitogenome was biased toward A and T, with 78.6% of A + T content (A = 39.2%, T = 39.4%, C = 12.6%, and G = 8.9%). Among the protein-coding genes (PCGs), six genes took the start codon of ATG, five genes used ATT as start codon, while *COI* gene and *ND1* gene got ACG and TTG, respectively. The termination codon of these PCGs had three types (seven genes used *TAA*, *CYTB* gene used TAG, five genes used T + tRNA).

**Figure 1.**
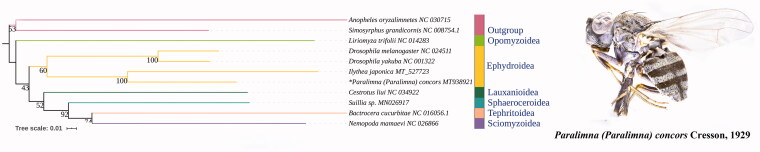
The phylogenetic tree of ML analysis based on 13PCGs and adult of *Paralimna (Paralimna) concors* Cression, 1929. ‘*’ Indicated new sequenced data in this study.

There are 10 species retrieved from NCBI and one new sequenced datum used in phylogenetic analysis. The genbank accession numbers are listed as follows: *Anopheles oryzalimnetes* NC_030715, *Bactrocera cucurbitae* NC_016056.1, *Ceratitis capitata* NC_000857, *Drosophila melanogaster* NC_024511, *Drosophila yakuba* NC_001322, *Ilythea japonica* MT_527723, *Liriomyza trifolii* NC_014283, *Nemopoda mamaevi* NC_026866, *Paralimna (Paralimna) concors MT938921, *Simosyrphus grandicornis* NC 008754.1, *Suillia* sp. MN026917. Thirteen PCGs were used to reconstruct phylogenetic relationship with the maximum likelihood method using IQTREE Web Server (http://iqtree.cibiv.univie.ac.at/) (Jana et al. 2016). The topology was given and bootstrap support numbers are shown in Figure 1. ML analysis revealed that *Ephydridae* was monophyletic. The higher-level relationship of Opomyzoidea + (Ephydroidea + (Lauxanioidea + (Sphaeroceroidea + (Sciomyzoidea + Tephritoidea)))) was supported.

The complete mitochondrial genome of *Paralimna (Paralimna) concors* provides valuable information for future genetic and evolutionary studies of family *Ephydridae* and superfamily Ephydroidea.

## Data Availability

The data that support the findings of this study are openly available in [NCBI] at [https://www.ncbi.nlm.nih.gov/], reference number [MT938921].

## References

[CIT0001] Bolger AM, Lohse M, Usadel B. 2014. Trimmomatic: a flexible trimmer for Illumina sequence data. Bioinformatics. 30(15):2114–2120. 24695404 10.1093/bioinformatics/btu170 PMC4103590

[CIT0002] Foote B. 1995. Biology of shore flies. Annu Rev Entomol. 40(1):417–442.

[CIT0403] Gillett, C.P., Cramptonplatt, A., Timmermans, M.J., Jordal, B.H., Emerson, B.C., Vogler, A.P. 2014. Bulk de novo mitogenome assembly from pooled total DNA elucidates the phylogeny of weevils (Coleoptera: Curculionoidea). Molecular Biology Evolution 31(8):2223–2237. 10.1093/molbev/msu154 PMC4104315 24803639

[CIT0003] Jana T, Lam-Tung N, Arndt, von H, Quang MB. 2016. W-IQ-TREE: a fast online phylogenetic tool for maximum likelihood analysis. Nuclc Acids Res. 44(W1):W232–W235. 10.1093/nar/gkw256 PMC4987875 27084950

[CIT0004] Krivosheina MG, Ozerov AL. 2020. A new species of the shore-fly genus Paralimna Loew, 1862 (Diptera: *Ephydridae*) from Australia. Russian Entomol J. 29(2):222–226.

[CIT0005] Li X, Wang Y, Su S, Yang D. 2016. The complete mitochondrial genomes of *Musca domestica* and *Scathophaga stercoraria* (Diptera: Muscoidea: Muscidae and Scathophagidae). Mitochondrial DNA Part A. 27(2):1435–1436. 10.3109/19401736.2014.953080 25163032

[CIT0006] Loew H. 1862. Monographs of the Diptera of North America. Part 1 Smithsonian Institution. Smithsonian Miscellan Collec. 6(141):1–221.

[CIT0007] Lowe TM, Chan PP. 2016. tRNAscan-SE on-line: integrating search and context for analysis of transfer RNA genes. Nucleic Acids Res. 44(W1):W54–W57. 27174935 10.1093/nar/gkw413 PMC4987944

[CIT0008] Mathis W, Zatwarnicki T. 1998. Family 3.49 *Ephydridae*. In: Papp L, Darvas B, editors. Contributions to a manual of Palaearctic Diptera, Vol. 3. Budapest, Hungary: Science Herald; p. 537–570.

[CIT0009] Pape T, Blagoderov V, Mostovski MB. 2011. Order Diptera Linnaeus, 1758. Zootaxa. 3148(1):222–229.

[CIT0010] Peng Y, Leung HCM, Yiu SM, Chin FYL. 2012. IDBA-UD: a de novo assembler for single-cell and metagenomic sequencing data with highly uneven depth. Bioinformatics. 28(11):1420–1428. 22495754 10.1093/bioinformatics/bts174

[CIT0011] Qilemoge , Gao S, Tang C, Wang N, Yang D. 2018. The mitochondrial genome of *Diostracus lamellatus* (Diptera: Dolichopodidae). Mitochondrial DNA Part B. 3(1):346–347. 33537413 10.1080/23802359.2018.1450662 PMC7831357

[CIT0012] Ren J, Yang Q, Gao S, Pan Z, Chang W, Yang D. 2019. The mitochondrial genome of *Limonia phragmitidis* (Diptera Limoniidae). Mitochondrial DNA Part B. 4(1):719–720.

[CIT0013] Zhou Q, Ding S, Li X, Zhang T, Yang D. 2017. Complete mitochondrial genome of *Allognosta vagans* (Diptera, Stratiomyidae). Mitochondrial DNA Part B. 2(2):461–462. 33473862 10.1080/23802359.2017.1357450 PMC7800880

